# Analysis of simultaneous multielectrode recordings with 4,096 channels: changing dynamics of spontaneous activity in the developing retina

**DOI:** 10.1186/1471-2202-12-S1-P296

**Published:** 2011-07-18

**Authors:** Matthias H Hennig, Alessandro Maccione, Mauro Gandolfo, Matthew Down, Stephen J Eglen, Luca Berdondini, Evelyne Sernagor

**Affiliations:** 1 Institute for Adaptive and Neural Computation, School of Informatics, University of Edinburgh, Edinburgh, EH8 9AB, UK; 2 Department of Neuroscience and Brain Technologies, Italian Institute of Technology, 16163 Genova, Italy; 3 Department of Biophysical and Electronic Engineering, University of Genova, 16145 Genova, Italy; 4 Institute of Neuroscience, Newcastle University Medical School, Newcastle upon Tyne, NE1 7RU, UK; 5 Department of Applied Mathematics and Theoretical Physics, Cambridge University, Cambridge, CB3 0WA, UK

## 

Our current understanding of the dynamics of neural circuits is limited by the poor resolution of multi-neuron recordings from large neural populations, which largely prevents the experimental verification of theoretical models and predictions. It is, for instance, difficult to distinguish between different potential classes of network architecture, such as feed-forward or recurrent networks, on the basis of simultaneous recordings from just tens of neurons. Recent advances in electronics have now made it possible to simultaneously record from thousands of neurons. Here, we used the Active Pixel Sensor (APS) multielectrode array (MEA) [1,2], consisting of 4,096 electrodes recording at near cellular resolution (21μm electrode diameter, 42μm centre-to-centre separation, arranged on a 64x64 lattice), to record spontaneous neural activity in the developing neonatal mouse retina *in vitro*. This activity takes the form of spontaneous propagating waves, which can be recorded in the ganglion cell layer [3,4]. This spontaneous activity occurs before the retina responsive to visual stimulation, and is thought to provide cues instructive for the wiring of visual connections. So far, retinal waves have been investigated with MEAs ranging from 60 [reviewed in ref. 4] to 512 electrodes [5], and with Ca^2+^ imaging [reviewed in refs. 3 and 4]. These previous studies were, therefore, either limited by the spatial resolution of the MEA, or by the slow temporal response of Ca^2+^ indicators. While it is well established that the properties of retinal waves change during development, so far wave dynamics have been extrapolated from these limited data sets. Here, we provide a complete characterisation of the dynamics of retinal waves during the first two postnatal weeks, and present several methods for the analysis of such activity patterns. In the mammalian retina, the earliest waves propagate through gap junctions (Stage I, prenatal in mouse), followed by lateral propagation between cholinergic starburst amacrine cells (Stage II) and finally by activity that depends on glutamatergic synaptic transmission (Stage III). Consistent with an earlier analysis of 60 channel MEA recordings [6], we found that Stage II waves exhibit a high degree of randomness with respect to initiation points, trajectories , sizes and durations. Stage III waves, on the other hand, were significantly faster and they were more restricted spatially, following several clear repetitive, non-random propagation patterns that appear to tile the retina, mostly starting from the periphery and propagating towards the centre. This latter effect can not be identified in recordings with conventional 60 channel MEAs, underscoring the importance of probing and analysing neural circuits at a near-cellular resolution.
